# Correlation of IVC Diameter and Collapsibility Index With Central Venous Pressure in the Assessment of Intravascular Volume in Critically Ill Patients

**DOI:** 10.7759/cureus.1025

**Published:** 2017-02-12

**Authors:** Abid Ilyas, Wasib Ishtiaq, Salman Assad, Haider Ghazanfar, Salman Mansoor, Muhammad Haris, Aayesha Qadeer, Aftab Akhtar

**Affiliations:** 1 Internal Medicine, Shifa College of Medicine, Islamabad, Pakistan; 2 Department of Pulmonology & Critical Care Medicine, Shifa International Hospital, Islamabad, Pakistan; 3 Department of Medicine, Shifa Tameer-e-Millat University, Islamabad, Pakistan; 4 Department of Neurology, Shifa International Hospital, Islamabad, Pakistan; 5 Department of Cardiology, Shifa College of Medicine, Islamabad, Pakistan

**Keywords:** collapsibility index, intensive care units, ultrasonography, inferior vena cava, central venous pressure, critical care

## Abstract

**Objective:**

The objective of our study is to assess the correlation between inferior vena cava (IVC) diameters, central venous pressure (CVP) and the IVC collapsibility index for estimating the volume status in critically ill patients.

**Methods:**

This cross-sectional study used the convenient sampling of 100 adult medical intensive care unit (ICU) patients for a period of three months. Patients ≥ 18 years of age with an intrathoracic central venous catheter terminating in the distal superior vena cava connected to the transducer to produce a CVP waveform were included in the study. A Mindray diagnostic ultrasound system model Z6 ultrasound machine (Mindray, NJ, USA) was used for all examinations. An Ultrasonic Transducer model 3C5P (Mindray, NJ, USA) for IVC imaging was utilized. A paired sampled t-test was used to compute the p-values.

**Results:**

A total of 32/100 (32%) females and 68/100 (68%) males were included in the study with a mean age of 50.4 ± 19.3 years. The mean central venous pressure maintained was 10.38 ± 4.14 cmH2O with an inferior vena cava collapsibility index of 30.68 ± 10.93. There was a statistically significant relation among the mean CVP pressure, the IVC collapsibility index, the mean maximum and minimum IVC between groups as determined by one-way analysis of variance (ANOVA) (p < 0.001). There was a strong negative correlation between CVP and IVC collapsibility index (%), which was statistically significant (r = -0.827, n = 100, p < 0.0005). A strong positive correlation between CVP and maximum IVC diameter (r = 0.371, n = 100, p < 0.0005) and minimum IVC diameter (r = 0.572, n = 100, p < 0.0005) was found.

**Conclusion:**

There is a positive relationship of CVP with minimum and maximum IVC diameters but an inverse relationship with the IVC collapsibility index.

## Introduction

Bedside assessment of intravascular volume status in critically ill patients is challenging. Fluid management impacts systemic perfusion and may influence the risk of organ failure and mortality [1]. Clinicians often use invasive hemodynamic monitoring as an adjunct to information gathered from the physical examination and laboratory evaluation to arrive at a fluid management strategy. Central venous pressure (CVP) is a hemodynamic parameter that is extensively used. A non-invasive and economical technique like ultrasound in the ICU helps to approach diagnosis and treatment of the critically ill patients [2]. A survey carried out in Canada concluded that 90% of the intensivists use CVP to monitor fluid resuscitation in septic shock patients [3]. High CVP is known to be associated with volume overload states while low CVP is associated with volume depleted states. CVP is a good approximation of right atrial pressure (RAP) which in turn is a major determinant to right ventricular filling. Therefore CVP is a good indicator of right ventricular preload. The complication associated with CVP insertion includes failure to place the catheter, arterial puncture, catheter malposition, pneumothorax, subcutaneous hematoma, hemothorax, asystolic cardiac arrest and catheter-related infection [4-5]. Bedside ultrasound is potentially a useful non-invasive adjunct to estimate the intravascular status by measuring IVC diameter [6-8]. One technique uses the size and collapsibility of the inferior vena cava (IVC), similar to the method used by echocardiographers to estimate right atrial pressure (RAP) in non-acute care settings. Cyclic changes in thoracic pressure in a healthy person may result in the collapse of approximately 50% of the IVC diameter [9]. Collapsibility of the inferior vena cava has been found to be useful in monitoring an acute heart failure patient’s response to therapy as well as assisting in ongoing resuscitation by providing means to measure CVP non-invasively [10-11].

In previous studies a head-to-head comparison has been made in spontaneously breathing patients to evaluate how well CVP was predicted by maximal IVC diameter and collapsibility with inspiration, hypothesizing that the IVC collapsibility index would have a superior predictive value for a CVP  > 10 mmHg than the maximal IVC diameter. The diameter variations of the vena cava can be of range 13–28 mm and mean 20 mm. There was no significant relation of vena cava diameters to height, weight, or body surface area based on previous studies. Vena cava diameters are well reproducible, with an interobserver error, estimated as the coefficient of variation of 2.2% (r = 0.98, p < 0.05) [12]. The rationale of our study is to assess correlation between the inferior vena cava (IVC) diameters, central venous pressure (CVP) and inferior vena cava collapsibility index for estimating the volume status in critically-ill patients.

## Materials and methods

This cross-sectional analysis used a convenient sampling of 100 adult medical intensive care unit (ICU) patients over a period of three months. Patients ≥ 18 years of age with an intrathoracic central venous catheter terminating in the distal superior vena cava connected to the transducer to produce a CVP waveform were included in the study. Patients with clinical signs of elevated abdominal pressure, moderate to severe tricuspid regurgitation, CVP inserted for more than 24 hours, and patients in whom the supine position was contraindicated were not included in the study.

The study was performed from January 2016 to May 2016 with a total duration of five months. Using the World health Organization (WHO) sample size calculator, keeping 95% confidence level and a prevalence of hypovolemia at 12.36%, a sample size of 85 was calculated. Informed consent was obtained from all the participants, and they were assured that the identity of the respondents will be kept anonymous. Ethical approval was obtained from the Shifa International Hospital ethical review board. Three critical care fellows prospectively enrolled eligible patients and completed the ultrasound examination of the IVC diameter. All three ultrasonographers had familiarity with bedside vascular ultrasound. Before the study began, they completed two hours of standardized training in image acquisition according to the study protocol. It was followed by five practice examinations under the critical care consultant and the supervisors.

During enrollment and collection of ultrasound data, study ultrasonographers were blinded to CVP monitoring. Bedside ultrasound images were obtained in a systematic fashion with the patient supine to determine the dimensions and collapsibility of the IVC. A Mindray diagnostic ultrasound system model Z6 ultrasound machine (Mindray, NJ, USA) was used for all examinations. Ultrasonographers used an Ultrasonic Transducer model 3C5P for IVC imaging (Mindray, NJ, USA). First, ultrasound gel was applied to the subxiphoid region. The IVC was imaged in a longitudinal plane with the transducer in the subxiphoid position. The intrahepatic segment of the IVC was visualized as it entered the right atrium. The IVC diameter was measured 2 cm caudal to the hepatic vein-IVC junction, or approximately 3–4 cm from the junction of the IVC and right atrium. This measurement location was preferred as IVC collapsibility in the intrahepatic segment was not influenced by the activity of the muscular diaphragm compared to one at the IVC-right atrial junction. 15 M-mode was used to capture a 10-s cine loop of the IVC over two or three respiratory cycles. The maximum IVC diameter (IVCdmax) was measured as the maximum anterior-posterior dimension at end-expiration using the leading edge technique (inner edge to inner edge of the vessel wall). In addition, the minimum IVC diameter was measured at end-inspiration (IVCdmin). The IVC collapsibility index was the difference between the maximum and minimum IVC diameters divided by the maximum IVC diameter, expressed as a percentage ([IVCdmax – IVCdmin] / IVCdmax × 100%).

Immediately following the ultrasound image acquisition, study personnel obtained a simultaneous recording of the CVP waveform from the distal lumen of the central venous catheter and a single-lead electrocardiogram rhythm strip. The CVP was uniformly measured from a recording at end expiration with the patient supine and the pressure transducer having been zeroed at the mid-thoracic position. A patient with CVP of less than 8 cmH2O was considered as hypovolemic. The patients with CVP between 8–12 cmH2O were considered as euvolemic and patients having CVP > 12 cmH2O were considered as hypervolemic. The data was entered and analyzed on SPSS version 21 (IBM, NY, USA). Descriptive statistics were calculated for both qualitative variables. One-way analysis of variance (ANOVA) was used for comparison between the three groups of patients with different intravascular volume status and Tukey's method was used for multiple comparisons. Pearson correlation coefficient was used to assess the significance between CVP and IVC collapsibility index (%) and the maximum and minimum IVC diameter. A p-value less than 0.05 was considered to be significant.

## Results

A total of 32/100 (32%) females and 68/100 (68%) males were included in the study with a mean age of 50.4 ± 19.3 years. This is presented in Table 1.

**Table 1:** Demographics

Age of the Patient (Mean ± Standard Deviation)	50.4±19.3 Years
Male Participants	68/100 (68%)
Female Participants	32/100 (32%)
On Invasive Ventilation	47/100 (47%)
Hypovolemic Group	26/100 (26%)
Euvolemic Group	46/100 (48%)
Hypervolemic Group	26/100 (26%)

The mean arterial pressure maintained was 82.6 ± 21.1 mmHg and mean positive end-expiratory pressure (PEEP) was 5.3 ± 1.3 cmH2O. The mean heart rate was 95.2 ± 21.1 per minute. The mean central venous pressure maintained was 10.38 ± 4.14 cmH2O with the inferior vena cava collapsibility index of 30.68 ± 10.93. The central venous pressure (CVP) was found to be less than 8 cmH2O among 26/100 (26%) patients, while 46/100 (48%) had CVP between 8–12 cmH2O and 26/100 (26%) patients had CVP greater than 12 cmH20. Invasive ventilation was done among 47/100 (47%) patients. The mean inferior vena cava (IVC) minimum diameter was 1.17 ± 0.27 cm and maximum diameter was 1.75 ± 0.27 cm.

One-way ANOVA test was used for comparison between the three groups of patients with different intravascular volume status. Tukey's method was used for multi-comparison. There was a statistically significant correlation in the mean CVP pressure and the IVC collapsibility index and the CVP with mean maximum and mean minimum IVC diameters between groups as determined by one-way ANOVA (p < 0.001). This is presented in Table 2.

**Table 2:** Parameter assessment with volume status *Hypovolemia CVP < 8 cmH2O, **Euvolemia CVP 8-12 cmH2O, ***Hypervolemia CVP > 12 cm H2O.

Parameters	Hypovolemia* n=26	Euvolemia** n=48	Hypervolemia*** n=26	Anova (p-value)
Mean Arterial Pressure (mmHg)	80.12±16.06	84.00±16.23	82.63±16.60	0.625
Heart Rate (per minute)	87.77±12.89	99.06±24.09	95.70±21.50	0.095
IVC Collapsibility Index	44.39± 8.05	30.21± 4.14	17.78± 2.69	0.001
Mean CVP Pressure (cmH2O)	5.42±1.63	10.06±1.48	15.73±2.11	0.001
IVC (Maximum Diameter) (centimeters)	1.63±0.19	1.68±0.16	1.78±0.16	0.006
IVC (Minimum Diameter) (centimeters)	0.94±0.17	1.15±0.21	1.42±0.24	0.001

A Tukey post hoc test revealed that the CVP and IVC minimum diameters were statistically lower in the hypovolemic group (p < 0.001) and statistically higher in the hypervolemia group (p < 0.001). It also revealed that IVC collapsibility index was statistically higher in the hypovolemic group and statistically lower in the hypervolemic group (p < 0.001). There was a significant difference in IVC (maximum diameter) between the hypovolemic and hypervolemic group (p = 0.004), as well as between the hypervolemic and euvolemic group (p = 0.025). There was no significant difference in IVC (maximum diameter) between the euvolemic and hypovolemic group (p=0.536). The IVC (minimum diameter) was statistically higher in the hypervolemic group (p < 0.001) and statistically lower in the hypovolemic group (p < 0.001). This is presented in Table 3.

**Table 3:** Comparison of mean arterial pressure, heart rate, IVC collapsibility index, mean CVP pressure, maximum and minimum IVC diameter of patients in the three groups of intravascular volume states using Tukey post-hoc test *Hypovolemia CVP < 8 cmH2O, **Euvolemia CVP 8-12 cmH2O, *** Hypervolemia CVP > 12 cm H2O.

Parameter	Volume Status	Mean ± SD	Tukey post-hoc test
Mean CVP Pressure (cmH2O)	Hypovolemia*	5.42±1.63	p<0.001
	Euvolemia**	10.06±1.48	
	Euvolemia**	10.06±1.48	p<0.001
	Hypervolemia***	15.73±2.11	
	Hypovolemia*	5.42±1.63	p>0.05
	Hypervolemia***	15.73±2.11	
IVC (Minimum Diameter) (centimeters)	Hypovolemia*	0.94±0.17	p<0.001
	Euvolemia**	1.15±0.21	
	Hypervolemia***	1.42±0.24	p<0.001
	Euvolemia**	1.15±0.21	
IVC Collapsibility Index	Hypovolemia*	44.39± 8.05	p<0.001
	Euvolemia**	30.21± 4.14	
	Euvolemia**	30.21± 4.14	p<0.001
	Hypervolemia***	17.78± 2.69	
IVC (Maximum Diameter) (centimeters)	Hypovolemia*	1.63±0.19	p<0.001
	Hypervolemia***	1.78±0.16	
	Euvolemia**	1.68±0.16	p<0.001
	Hypervolemia***	1.78±0.16	

**Figure 1:**
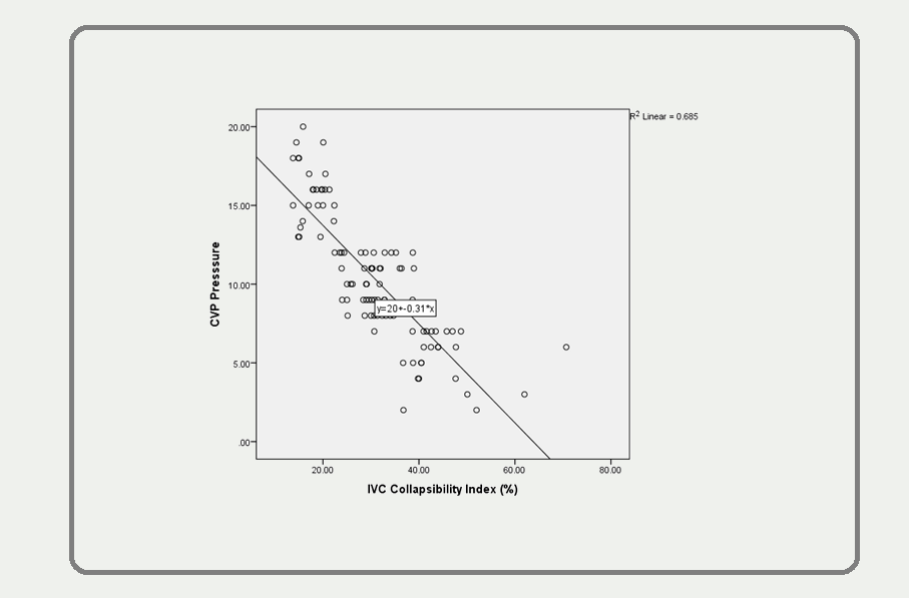
A strong positive correlation was revealed between CVP and maximum IVC diameter (r = 0.371, n = 100, p

A Pearson correlation was run to determine the relationship between the central venous pressure values and the inferior vena cava collapsibility index (%) and the maximum and minimum inferior vena cava diameter. A strong negative linear correlation was observed between the central venous pressure (10.38 ± 4.14 cmH2O) and the inferior vena cava collapsibility index (%) (30.68 ± 10.93), which was statistically significant (r = -0.827, p < 0.0005). A strong positive correlation was revealed between the central venous pressure (10.38 ± 4.14 cmH2O) and the maximum inferior vena cava diameter (1.75 ± 0.27 cm) (r = 0.371, n = 100, p < 0.0005) and the minimum IVC diameter (1.17 ± 0.27 cm) (r = 0.572, n = 100, p<0.0005). This is presented in Figure 1.

## Discussion

The indication of CVP measurement included diagnostic measurement and monitoring. This helped in fluid management and pharmacological intervention [13]. Although CVP monitoring is a useful tool for guiding fluid management and monitoring, it requires placement of a central venous catheter, which is an invasive procedure and is associated with complications. Bedside sonography has emerged as a potentially useful tool for noninvasive assessment of the intravascular fluid status by measuring the IVC diameter.

Initially, intravascular volume status assessed by the inferior vena cava ultrasound (IVC-US) was focused on comparing the IVC diameter (size) with the measured CVP. The outcomes of these studies suggested a positive correlation of the mean IVC diameter with the central venous pressure (CVP) [14-16]. These results are comparable to our findings. Further studies have compared CVP with ultrasonographic measurements of IVC respirophasicity, rather than IVC diameter alone to assess the dynamic markers of intravascular volume. These studies conclude that there is an inverse relationship of the collapsibility index (CI) to the CVP at extremes of intravascular fluid volume. In a small group of patients with suspected sepsis, Nagdev, et al. [7] reported that CI > 50% had a strong association with a lower CVP < 8 mmHg. Kircher, et al. [17], reported the similar results that collapsibility index (CI) > 50% was associated with lower right atrial (RA) pressures < 10 mmHg, whereas CI < 50% indicated raised RA pressures more than 10 mmHg. Brennen, et al. documented that the combination of both collapsibility indices (CI) and IVC diameter measurements may assist in improved ultrasonographic evaluations of the IVC with clinically important categories of right atrial pressure (e.g. 0–10 mmHg); however, the limitation of the study was the exclusion of ventilated patients and poor statistical significance that limits the applicability of this approach to our investigation of predominately critically ill patients [16].

Stawicki SP, et al. [4] demonstrated that the collapsibility index (CI) strongly correlates with low (< 20%) and high (> 60%) CVP values and suggested that the closer the CI is to 0% or 100%, the more is the probability that the patient is either volume-overloaded or volume-depleted, respectively. There is no such evidence that clearly supports a linear relationship between CI and CVP; however, there is an inverse relationship of CVP with CI when CI values are either very high or low. The ability to predict CVP values precisely is of untested clinical gain, keeping in view the poor performance of CVP as a marker of intravascular volume and fluid responsiveness. A very high CI (often associated with a very low CVP) may serve as a rational sign that it is harmless to give more fluid without volume overload. The decrease of CI with fluid administration makes it a less reliable substitute for intravascular volume. Thanakitcharu P, et al. [10] supported the correlation between central venous pressure (CVP) and IVC collapsibility indices (IVC-CI). The authors conclude that the IVC-CI can provide a useful guide for noninvasive intravascular volume status assessment in critically ill patients.

## Conclusions

There is a positive relationship of CVP with minimum and maximum IVC diameters but an inverse relation with IVC collapsibility index. A very high CI (often associated with a very low CVP) may serve as a possible explanation for the beneficial use of giving more fluid without volume overload. As the CI decreases with fluid administration, it becomes increasingly less reliable as a surrogate for intravascular volume.
